# Sample size, power and effect size revisited: simplified and practical approaches in pre-clinical, clinical and laboratory studies

**DOI:** 10.11613/BM.2021.010502

**Published:** 2020-12-15

**Authors:** Ceyhan Ceran Serdar, Murat Cihan, Doğan Yücel, Muhittin A Serdar

**Affiliations:** 1 Medical Biology and Genetics, Faculty of Medicine, Ankara Medipol University, Ankara, Turkey; 2 Ordu University Training and Research Hospital, Ordu, Turkey; 3 Department of Medical Biochemistry, Lokman Hekim University School of Medicine, Ankara, Turkey; 4 Department of Medical Biochemistry, Acibadem Mehmet Ali Aydinlar University, Istanbul, Turkey

**Keywords:** biostatistics, effect size, power analysis, sample size

## Abstract

Calculating the sample size in scientific studies is one of the critical issues as regards the scientific contribution of the study. The sample size critically affects the hypothesis and the study design, and there is no straightforward way of calculating the effective sample size for reaching an accurate conclusion. Use of a statistically incorrect sample size may lead to inadequate results in both clinical and laboratory studies as well as resulting in time loss, cost, and ethical problems. This review holds two main aims. The first aim is to explain the importance of sample size and its relationship to effect size (ES) and statistical significance. The second aim is to assist researchers planning to perform sample size estimations by suggesting and elucidating available alternative software, guidelines and references that will serve different scientific purposes.

## Introduction

Statistical analysis is a crucial part of a research. A scientific study must include statistical tools in the study, beginning from the planning stage. Developed in the last 20-30 years, information technology, along with evidence-based medicine, increased the spread and applicability of statistical science. Although scientists have understood the importance of statistical analysis for researchers, a significant number of researchers admit that they lack adequate knowledge about statistical concepts and principles (*1*). In a study by West and Ficalora, more than two-thirds of the clinicians emphasized that “the level of biostatistics education that is provided to the medical students is not sufficient” (*2*). As a result, it was suggested that statistical concepts were either poorly understood or not understood at all (*3*, *4*). Additionally, intentionally or not, researchers tend to draw conclusions that cannot be supported by the actual study data, often due to the misuse of statistics tools (*5*). As a result, a large number of statistical errors occur affecting the research results.

Although there are a variety of potential statistical errors that might occur in any kind of scientific research, it has been observed that the sources of error have changed due to the use of dedicated software that facilitates statistics in recent years. A summary of main statistical errors frequently encountered in scientific studies is provided below (*6*-*13*):

Flawed and inadequate hypothesis;
Improper study design;
Lack of adequate control condition/group;
Spectrum bias;
Overstatement of the analysis results;
Spurious correlations;
Inadequate sample size;
Circular analysis (creating bias by selecting the properties of the data retrospectively);
Utilization of inappropriate statistical studies and fallacious bending of the analyses;
p-hacking (*i.e.* addition of new covariates *post hoc* to make P values significant);
Excessive interpretation of limited or insignificant results (subjectivism);
Confusion (intentionally or not) of correlations, relationships, and causations;
Faulty multiple regression models;
Confusion between P value and clinical significance; and
Inappropriate presentation of the results and effects (erroneous tables, graphics, and figures).

## Relationship among sample size, power, P value and effect size

**Figure 1:**
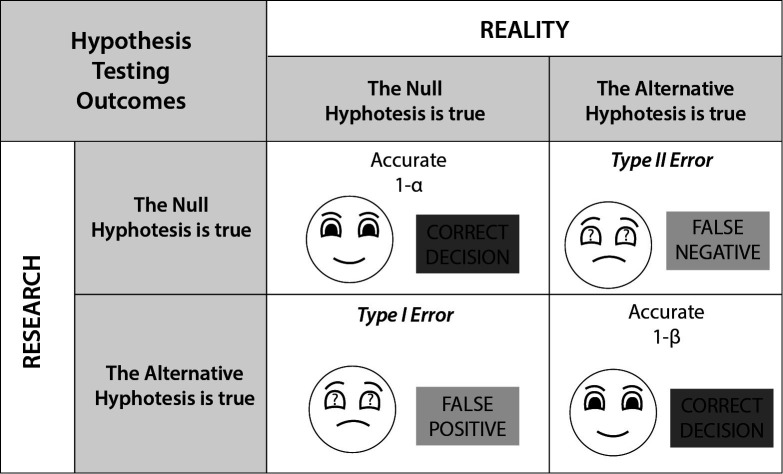
Illustration of Type I and Type II errors.

In this review, we will concentrate on the problems associated with the relationships among sample size, power, P value, and effect size (ES). Practical suggestions will be provided whenever possible. In order to understand and interpret the sample size, power analysis, effect size, and P value, it is necessary to know how the hypothesis of the study was formed. It is best to evaluate a study for Type I and Type II errors (Figure 1) through consideration of the study results in the context of its hypotheses (*14*-*16*).

A statistical hypothesis is the researcher’s best guess as to what the result of the experiment will show. It states, in a testable form the proposition the researcher plans to examine in a sample to be able to find out if the proposition is correct in the relevant population. There are two commonly used types of hypotheses in statistics. These are the null hypothesis (H0) and the alternative (H1) hypothesis. Essentially, the H1 is the researcher’s prediction of what will be the situation of the experimental group after the experimental treatment is applied. The H0 expresses the notion that there will be no effect from the experimental treatment.

Prior to the study, in addition to stating the hypothesis, the researcher must also select the alpha (α) level at which the hypothesis will be declared “supported”. The α represents how much risk the researcher is willing to take that the study will conclude H1 is correct when (in the full population) it is not correct (and thus, the null hypothesis is really true). In other words, alpha represents the probability of rejecting H0 when it actually is true. (Thus, the researcher has made an error by reporting that the experimental treatment makes a difference, when in fact, in the full population, that treatment has no effect.)

The most common α level chosen is 0.05, meaning the researcher is willing to take a 5% chance that a result supporting the hypothesis will be untrue in the full population. However, other alpha levels may also be appropriate in some circumstances. For pilot studies, α is often set at 0.10 or 0.20. In studies where it is especially important to avoid concluding a treatment is effective when it actually is not, the alpha may be set at a much lower value; it might be set at 0.001 or even lower. Drug studies are examples for studies that often set the alpha at 0.001 or lower because the consequences of releasing an ineffective drug can be extremely dangerous for patients.

Another probability value is called “the P value”. The P value is simply the obtained statistical probability of incorrectly accepting the alternate hypothesis. The P value is compared to the alpha value to determine if the result is “statistically significant”, meaning that with high probability the result found in the sample will also be true in the full population. If the P value is at or lower than alpha, H1 is accepted. If it is higher than alpha, the H1 is rejected and H0 is accepted instead.

There are actually two types of errors: the error of accepting H1 when it is not true in the population; this is called a Type I error; and is a false positive. The alpha defines the probability of a Type I error. Type I errors can happen for many reasons, from poor sampling that results in an experimental sample quite different from the population, to other mistakes occurring in the design stage or implementation of the research procedures. It is also possible to make an erroneous decision in the opposite direction; by incorrectly rejecting H1 and thus wrongly accepting H0. This is called a Type II error (or a false negative). The β defines the probability of a Type II error. The most common reason for this type of error is small sample size, especially when combined with moderately low or low effect sizes. Both small sample sizes and low effect sizes reduce the power in the study.

Power, which is the probability of rejecting a false null hypothesis, is calculated as 1-β (also expressed as “1 - Type II error probability”). For a Type II error of 0.15, the power is 0.85. Since reduction in the probability of committing a Type II error increases the risk of committing a Type I error (and *vice versa*), a delicate balance should be established between the minimum allowed levels for Type I and Type II errors. The ideal power of a study is considered to be 0.8 (which can also be specified as 80%) (*17*). Sufficient sample size should be maintained to obtain a Type I error as low as 0.05 or 0.01 and a power as high as 0.8 or 0.9.

However, when power value falls below < 0.8, one cannot immediately conclude that the study is totally worthless. In parallel with this, the concept of “cost-effective sample size” has gained importance in recent years (*18*).

Additionally, the traditionally chosen alpha and beta error limits are generally arbitrary and are being used as a convention rather than being based on any scientific validity. Another key issue for a study is the determination, presentation and discussion of the effect size of the study, as will be discussed below in detail.

Although increasing the sample size is suggested to decrease the Type II errors, it will increase the cost of the project and delay the completion of the research activities in a foreseen period of time. In addition, it should not be forgotten that redundant samples may cause ethical problems (*19*, *20*).

Therefore, determination of the effective sample size is crucial to enable an efficient study with high significance, increasing the impact of the outcome. Unfortunately, information regarding sample size calculations are not often provided by clinical investigators in most diagnostic studies (*21*, *22*).

## Calculation of the sample size

**Figure 2:**
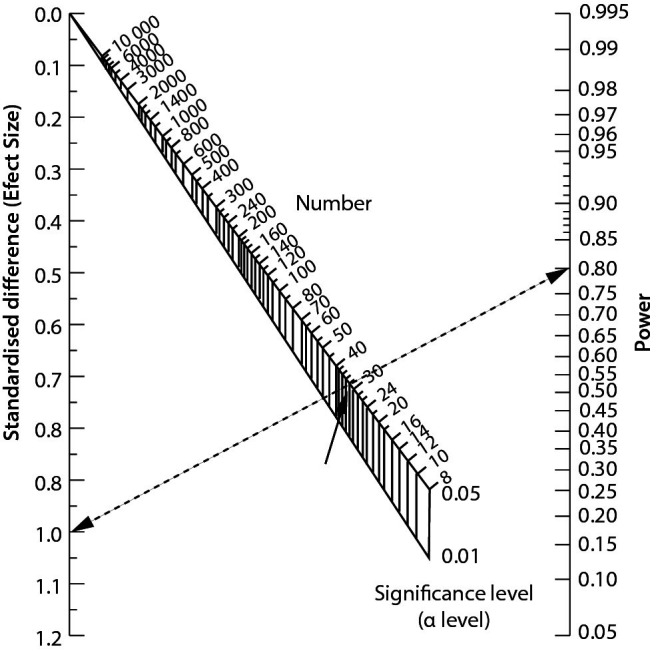
Nomogram for sample size and power, for comparing two groups of equal size. Gaussian distributions assumed. Standardized difference (effect size) and aimed power values are initially selected on the nomogram. The line connecting these values cross the significance level region of the nomogram. The intercept at the appropriate significance value presents the required sample size for the study. In the above example, for effect size = 1, power = 0.8 and alpha value = 0.05, the sample size is found to be 30. (Adapted from reference 16).

Different methods can be utilized before the onset of the study to calculate the most suitable sample size for the specific research. In addition to manual calculation, various nomograms or software can be used. The Figure 2 illustrates one of the most commonly used nomograms for sample size estimation using effect size and power (*23*).

Although manual calculation is preferred by the experts of the subject, it is a bit complicated and difficult for the researchers that are not statistics experts. In addition, considering the variety of the research types and characteristics, it should be noted that a great number of calculations will be required with too many variables (Table 1) (*16*, *24*-*30*).

**Table 1:** Sample size calculation formulas for some research methods (according to reference 17-23)

**Study type**	**Formulas**	**Explanations**
Proportion in survey type of studies	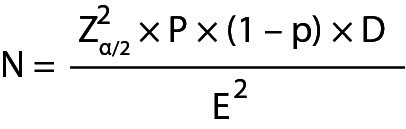	**N** - sample size
		**P** - prevalence or proportion of event
		**E** - precision (or margin of error) with which a researcher want to measure something
		**D** - design effect reflects the sampling design used in the survey type of study. This is 1 for simple random sampling and higher values (usually 1 to 2) for other designs such as stratified, systematic, cluster random sampling
		**Z_α/2_** - 1.96 for alpha 0.05
Group mean	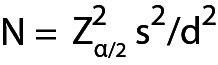	**s** - standard deviation obtained from previous study, or pilot study
		**d** - accuracy of estimate or how close to the true mean
		**Z_α/2_** -1.96 for alpha 0.05
**Study type**	**Formulas**	**Explanations**
Two means	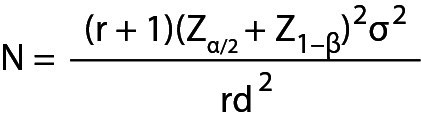	**r** = n1/n2 - the ratio of sample size
		**σ** - pooled standard deviation
		**d** - difference of means of 2 groups
		**Z_1-β_** - 0.84 for power 0.80
		**Z_α/2_** -1.96 for alpha 0.05
Two proportions	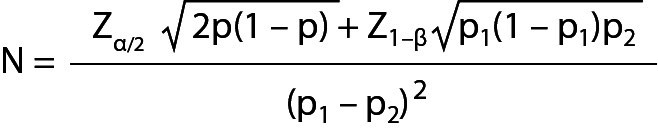	**Z_α/2_** -1.96 for alpha 0.05
		**Z_1-β_** - 0.84 for power 0.80
		**p1** and **p2** - proportion of event of interest (outcome) for group I and group II
		**p** - (p1+p2) / 2
Odds ratio	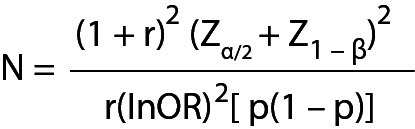	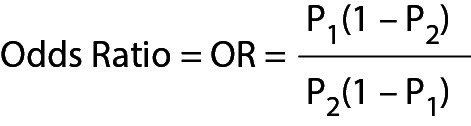
		**p1** and **p2** - proportion of event of interest (outcome) for group I and group II,
		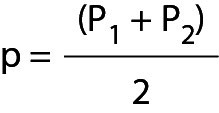
		**Z_α/2_** -1.96 for alpha 0.05
		**Z_1-β_** - 0.84 for power 0.80
Correlation coefficient	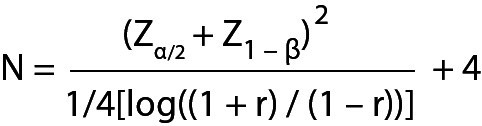	**r** - correlation between 2
		**Z_α/2_** -1.96 for alpha 0.05
		**Z_1-β_** - 0.84 for power 0.80
Diagnostic prognostic studies (ROC) analysis	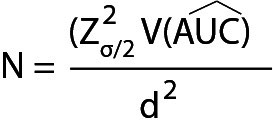 or	**AUC** - area under the curve
	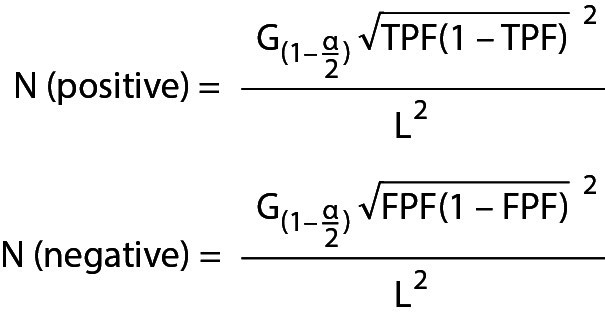	**L** - desired width of one half of the confidence interval
		**G_(1-α/2)_** – 1 - α/2 percentile of the standard normal distribution and α is the desired confidence level of the estimate
		**TPF** - true positive fraction, sensitivity
		**FPF** - false positive fraction
		**TNF** - true negative fraction, specificity
**Study type**	**Formulas**	**Explanations**
Adequate sensitivity/speciﬁcity	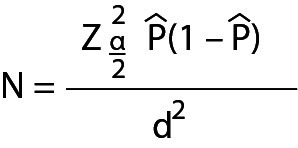	**P** - expected sensitivity
		**D** - allowable error
		**Z_α/2_** -1.96 for alpha 0.05
Questionnaire (Survey)	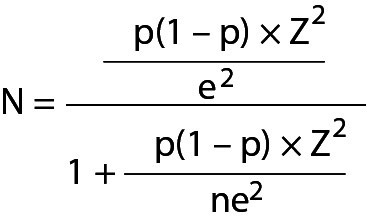 or	**N** - sample size
	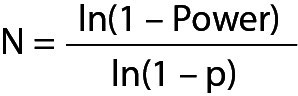	**n** - population size
		**p** - population proportion
		**e** - margin of error (percentage in decimal form)
		**z** - z-score

In recent years, numerous software and websites have been developed which can successfully calculate sample size in various study types. Some of the important software and websites are listed in Table 2 and are evaluated based both on the remarks stated in the literature and on our own experience, with respect to the content, ease of use, and cost (*31*, *32*). G-Power, R, and Piface stand out among the listed software in terms of being free-to use. G-Power is a free-to use tool that be used to calculate statistical power for many different t-tests, F-tests, χ^2^ tests, z-tests and some exact tests. R is an open source programming language which can be tailored to meet individual statistical needs, by adding specific program modules called packages onto a specific base program. Piface is a java application specifically designed for sample size estimation and *post-hoc* power analysis. The most professional software is PASS (Power Analysis and Sample Size). With PASS, it is possible to analyse sample size and power for approximately 200 different study types. In addition, many websites provide substantial aid in calculating power and sample size, basing their methodology on scientific literature.

**Table 2:** Software and websites that can be used for calculation of sample size and/or power analysis

**Programs**	**Performance**	**User** **Friendly**	**Freely** **available**	**Website**
G*Power	***	***	Yes	http://www.gpower.hhu.de
PS	**	***	Yes	http://biostat.mc.vanderbilt.edu/wiki/Main/PowerSampleSize
Piface	**	***	Yes	https://homepage.divms.uiowa.edu/~rlenth/Power/index.html
PASS	****	***	No	https://www.ncss.com/software/pass
nQuery	***	***	No	https://www.statsols.com/nquery-sample-size-and-power-calculation-for-successful-clinical-trials
R packages				
pwr	***	**	Yes	https://cran.r-project.org/web/packages/pwr
TrialSize	***	**	Yes	https://cran.r-project.org/web/packages/TrialSize
PowerUpR	***	**	Yes	https://cran.r-project.org/web/packages/PowerUpR
powerSurvEpi	***	**	Yes	https://CRAN.R-project.org/package=powerSurvEpi
SAS (PROC POWER)	****	***	No	https://support.sas.com/documentation/cdl/en/statug/63033/HTML/default/viewer.htm#power_toc.htm
SPSS (SamplePower)	***	***	No	https://www-01.ibm.com/marketing/iwm/iwmdocs/tnd/data/web/en_US/trialprograms/U741655I36057W80.html
STATA (power)	****	***	No	https://www.stata.com/features/power-and-sample-size/
Medcalc	*	****	No	https://www.medcalc.org/
Minitab	**	***	No	https://www.minitab.com/en-us/
**Programs**	**Performance**	**User** **Friendly**	**Freely** **available**	**Website**
Systat	***	****	No	https://systatsoftware.com/
Statistica	***	***	No	http://www.statsoft.com/Products/STATISTICA-Features
Microsoft Excel				
PowerUp	**	***	Yes	http://www.causalevaluation.org/power-analysis.html
XLSTAT	***	***	No	https://www.xlstat.com/en/
GenStat	**	***	No	https://genstat.kb.vsni.co.uk/videos/
Websites-Online				
Power and Sample Size	**	***	Yes	http://powerandsamplesize.com/Calculators/
StatCalc	**	***	Yes	https://www.cdc.gov/epiinfo/user-guide/statcalc/statcalcintro.html
Biomath	**	**	Yes	http://biomath.info/power/index.html
Openepi	**	***		https://www.openepi.com/SampleSize
UCSF Biostatistics	**	***	Yes	https://www.stat.ubc.ca/~rollin/stats/ssize/
Clincalc.com	*	***	Yes	https://clincalc.com/stats/samplesize.aspx
Sample Size Calculators	**	***	Yes	http://www.sample-size.net/
Genetic Power Calculator	***	**	Yes	http://zzz.bwh.harvard.edu/gpc/
OSSE, Sample Size Estimator (for SNPs)	*	***	Yes	http://osse.bii.a-star.edu.sg/
Surveys	**	**	Yes	https://surveysystem.com/sscalc.html http://www.raosoft.com/samplesize.html https://www.surveymonkey.com/mp/sample-size-calculator/

The sample size or the power of the study is directly related to the ES of the study. What is this important ES? The ES provides important information on how well the independent variable or variables predict the dependent variable. Low ES means that, independent variables don’t predict well because they are only slightly related to the dependent variable. Strong ES means that, independent variables are very good predictors of the dependent variable. Thus, ES is clinically important for evaluating how efficiently the clinicians can predict outcomes from the independent variables.

The scale of the ES values for different types of statistical tests conducted in different study types are presented in Table 3.

**Table 3:** Thresholds for interpreting the effect size

		**Effect Size (ES)**		
**Test**	**Relevant effect size**	**Small**	**Medium**	**Large**
t-test for means	Cohen’s d	0.2	0.5	0.8
Chi-Square	Cohen’s ω	0.1	0.3	0.5
r x c frequency tables	Cramer’s V or Phi	0.1	0.3	0.5
Correlation studies	*r*	0.2	0.5	0.8
2 x 2 table case control	Odd Ratio (OR)	1.5	2	3
2 x 2 table cohort studies	Risk Ratio (RR)	2	3	4
One-way an(c)ova (regression)	Cohen’s f	0.1	0.25	0.4
ANOVA (for large sample)	Eta Square ɳ^2^	0.01	0.06	0.14
ANOVA (for small size)	Omega square Ω^2^			
Friedman test	Average spearman Rho	0.1	0.3	0.5
Multiple regression	ɳ^2^	0.02	0.13	0.26
Coefficient of determination	r^2^	0.04	0.25	0.64
Number needed to treat	NNT	1 / Initial risk		

In order to evaluate the effect of the study and indicate its clinical significance, it is very important to evaluate the effect size along with statistical significance. P value is important in the statistical evaluation of the research. While it provides information on presence/absence of an effect, it will not account for the size of the effect. For comprehensive presentation and interpretation of the studies, both effect size and statistical significance (P value) should be provided and considered.

It would be much easier to understand ES through an example. For example, assume that independent sample t-test is used to compare total cholesterol levels for two groups having normal distribution. Where X, SD and N stands for mean, standard deviation and sample size, respectively. Cohen’s d ES can be calculated as follows:

**Mean (X), mmol/L Standard deviation (SD) Sample size (N)**

Group 1 6.5 0.5 30

Group 2 5.2 0.8 30

Pooled standard deviation (SDp) = √((SD_group1_^2^) + (SD_group2_^2^) / 2 = √((0.5^2^) + (0.8^2^)) / 2 = √0.445 = 0.67 (Equation (Eq.) 1)

Degrees of freedom (DF) = (N_group1_ – 1) + (N_group2_ – 1) = (30 – 1) + (30 – 1) = 58 (Eq. 2)

t value= - 7.54, P < 0.001 Cohen d ED = (X1 –X2)/SDp = (6.5-5.2) / 0.67 = 1.3 / 0.67 = 1.94 (Eq. 3)

Cohen d ES results represents: 0.8 large, 0.5 medium, 0.2 small effects). The result of 1.94 indicates a very large effect. Means of the two groups are remarkably different.

In the example above, the means of the two groups are largely different in a statistically significant manner. Yet, clinical importance of the effect (whether this effect is important for the patient, clinical condition, therapy type, outcome, *etc*.) needs to be specifically evaluated by the experts of the topic.

Power, alpha values, sample size, and ES are closely related with each other. Let us try to explain this relationship through different situations that we created using G-Power (*33*, *34*).

**Figure 3:**
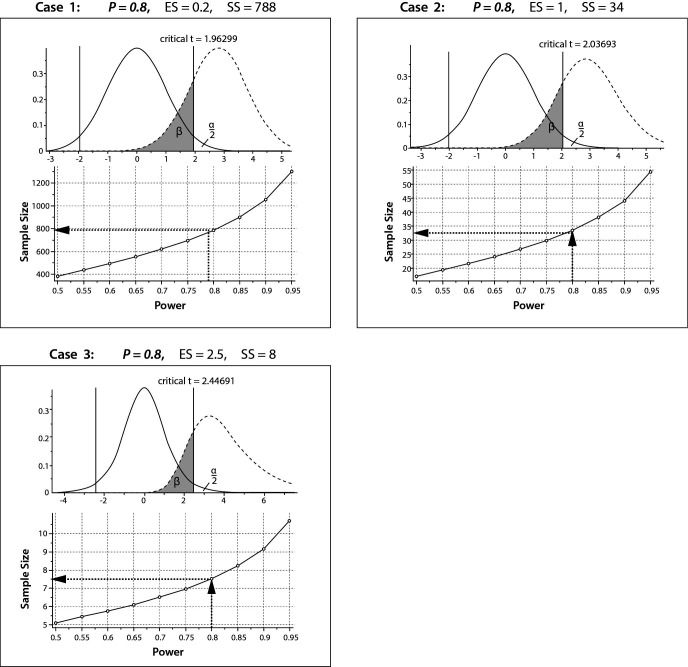
Relationship between effect size and sample size. P – power. ES - effect size. SS - sample size. The required sample size increases as the effect size decreases. In all cases, P value is set to 0.8. The sample sizes (SS) when ES is 0.2, 1, or 2.5; are 788, 34 and 8, respectively. The graphs at the bottom represent the influence of change in the sample size on the power.

The Figure 3 shows the change of sample size depending on the ES changes (0.2, 1 and 2.5, respectively) provided that the power remains constant at 0.8. Arguably, case 3 is particularly common in pre-clinical studies, cell culture, and animal studies (usually 5-10 samples in animal studies or 3-12 samples in cell culture studies), while case 2 is more common in clinical studies. In clinical, epidemiological or meta-analysis studies, where the sample size is very large; case 1, which emphasizes the importance of smaller effects, is more commonly observed (*33*).

**Figure 4:**
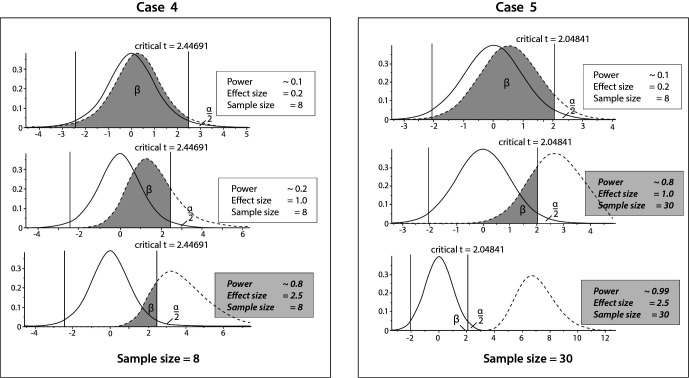
Relationship between effect size and power. Two different cases are schematized where the sample size is kept constant either at 8 or at 30. When the sample size is kept constant, the power of the study decreases as the effect size decreases. When the effect size is 2.5, even 8 samples are sufficient to obtain power = ~0.8. When the effect size is 1, increasing sample size from 8 to 30 significantly increases the power of the study. Yet, even 30 samples are not sufficient to reach a significant power value if effect size is as low as 0.2.

In Figure 4, case 4 exemplifies the change in power and ES values when the sample size is kept constant (*i.e.* as low as 8). As can be seen here, in studies with low ES, working with few samples will mean waste of time, redundant processing, or unnecessary use of laboratory animals.

Likewise, case 5 exemplifies the situation where the sample size is kept constant at 30. In this case, it is important to note that when ES is 1, the power of the study will be around 0.8. Some statisticians arbitrarily regard 30 as a critical sample size. However, case 5 clearly demonstrates that it is essential not to underestimate the importance of ES, while deciding on the sample size.

Especially in recent years, where clinical significance or effectiveness of the results has outstripped the statistical significance; understanding the effect size and power has gained tremendous importance (*35*–*38*).

Preliminary information about the hypothesis is eminently important to calculate the sample size at intended power. Usually, this is accomplished by determining the effect size from the results of a previous study or a preliminary study. There are software available which can calculate sample size using the effect size

We now want to focus on sample size and power analysis in some of the most common research areas.

## Determination of sample size in pre-clinical studies

Animal studies are the most critical studies in terms of sample size. Especially due to ethical concerns, it is vital to keep the sample size at the lowest sufficient level. It should be noted that, animal studies are radically different from human studies because many animal studies use inbred animals having extremely similar genetic background. Thus, far fewer animals are needed in the research because genetic differences that could affect the study results are kept to a minimum (*39*, *40*).

Consequently, alternative sample size estimation methodologies were suggested for each study type (*41*-*44*). If the effect size is to be determined using the results from previous or preliminary studies, sample size estimation may be performed using G-Power. In addition, Table 4 may also be used for easy estimation of the sample size (*40*).

**Table 4:** Cohen’s d for 4–34 samples *per* group assuming 0.8 and 0.9 power, a 0.05 significance level and a one-sided or two-sided test (Simplified from reference 40)

**Sample** **size**	**80%** **one-sided**	**90%** **one-sided**	**80%** **two-sided**	**90%** **two-sided**
**4**	2	2.35	2.38	2.77
**5**	1.72	2.03	2.02	2.35
**6**	1.54	1.82	1.8	2.08
**7**	1.41	1.66	1.63	1.89
**8**	1.31	1.54	1.51	1.74
**9**	1.23	1.44	1.41	1.63
**10**	1.16	1.36	1.32	1.53
**12**	1.05	1.23	1.2	1.39
**14**	0.97	1.14	1.1	1.27
**16**	0.9	1.06	1.02	1.18
**18**	0.85	1	0.96	1.11
**20**	0.8	0.94	0.91	1.05
**22**	0.76	0.9	0.86	1
**24**	0.73	0.86	0.83	0.96
**26**	0.7	0.82	0.79	0.92
**28**	0.67	0.79	0.76	0.88
**30**	0.65	0.76	0.74	0.85
**32**	0.63	0.74	0.71	0.82
**34**	0.61	0.72	0.69	0.8

In addition to sample size estimations that may be computed according to Table 4, formulas stated in Table 1 and the websites mentioned in Table 2 may also be utilized to estimate sample size in animal studies. Relying on previous studies pose certain limitations since it may not always be possible to acquire reliable “pooled standard deviation” and “group mean” values.

Arifin *et al.* proposed simpler formulas (Table 5) to calculate sample size in animal studies (*45*). In group comparison studies, it is possible to calculate the sample size as follows: N = (DF/k)+1 (Eq. 4).

**Table 5:** Sample size formulas for different types of group comparison studies (According to reference 45)

**Study design (Statistical test)**	**Minimum sample size / group**	**Maximum sample size / group**
Group comparison (ANOVA)	= (10 / k) + 1	= (20 / k) + 1
One group, repeated measures (one within factor, repeated measures ANOVA)	= 10 (r - 1) + 1^a,b^	= 20 (r - 1) + 1^a,b^
Group comparison, repeated measures (one-between, one within factor, repeated measures ANOVA)	= (10 / kr) + 1^b^	= (20 / kr) + 1^b^
k - number of groups. N - number of subjects *per* group. r - number of repeated measurements. a = N, because only one group is involved, b - must be multiplied by r whenever the experiment involves sacrificing the animals at each measurement.		

Based on acceptable range of the degrees of freedom (DF), the DF in formulas are replaced with the minimum (*10*) and maximum (*20*). For example, in an experimental animal study where the use of 3 investigational drugs are tested minimum number of animals that will be required: N = (10/3)+1 = 4.3; rounded up to 5 animals / group, total sample size = 5 x 3 = 15 animals. Maximum number of animals that will be required: N = (20/3)+1 = 7.7; rounded down to 7 animals / group, total sample size = 7 x 3 = 21 animals.

In conclusion, for the recommended study, 5 to 7 animals *per* group will be required. In other words, a total of 15 to 21 animals will be required to keep the DF within the range of 10 to 20.

In a compilation where Ricci *et al.* reviewed 15 studies involving animal models, it was noted that the sample size used was 10 in average (between 6 and 18), however, no formal power analysis was reported by any of the groups. It was striking that, all studies included in the review have used parametric analysis without prior normality testing (*i.e.* Shapiro-Wilk) to justify their statistical methodology (*46*).

It is noteworthy that, unnecessary animal use could be prevented by keeping the power at 0.8 and selecting one-tailed analysis over two-tailed analysis with an accepted 5% risk of making type I error as performed in some pharmacological studies, reducing the number of required animals by 14% (*47*).

Neumann *et al.* proposed a group-sequential design to minimize animal use without a decrease in statistical power. In this strategy, researchers started the experiments with only 30% of the animals that were initially planned to be included in the study. After an interim analysis of the results obtained with 30% of the animals, if sufficient power is not reached, another 30% is included in the study. If results from this initial 60% of the animals provide sufficient statistical power, then the rest of the animals are excused from the study. If not, the remaining animals are also included in the study. This approach was reported to save 20% of the animals in average, without leading to a decrease in statistical power (*48*).

Alternative sample size estimation strategies are implemented for animal testing in different countries. As an example, a local authority in southwestern Germany recommended that, in the absence of a formal sample size estimation, less than 7 animals *per* experimental group should be included in pilot studies and the total number of experimental animals should not exceed 100 (*48*).

On the other hand, it should be noted that, for a sample size of 8 to 10 animals *per* group, statistical significance will not be accomplished unless a large or very large ES (> 2) is expected (*45*, *46*). This problem remains as an important limitation for animal studies. Software like G-Power can be used for sample size estimation. In this case, results obtained from a previous or a preliminary study will be required to be used in the calculations. However, even when a previous study is available in literature, using its data for a sample size estimation will still pose an uncertainty risk unless a clearly detailed study design and data is provided in the publication. Although researchers suggested that reliability analyses could be performed by methods such as Markov Chain Monte Carlo, further research is needed in this regard (*49*).

The output of the joint workshop held by The National Institutes of Health (NIH), Nature Publishing Group and Science; “Principles and Guidelines for Reporting Preclinical Research” that was published in 2014, has since been acknowledged by many organizations and journals. This guide has shed significant light on studies using biological materials, involving animal studies, and handling image-based data (*50*).

**Figure 5:**
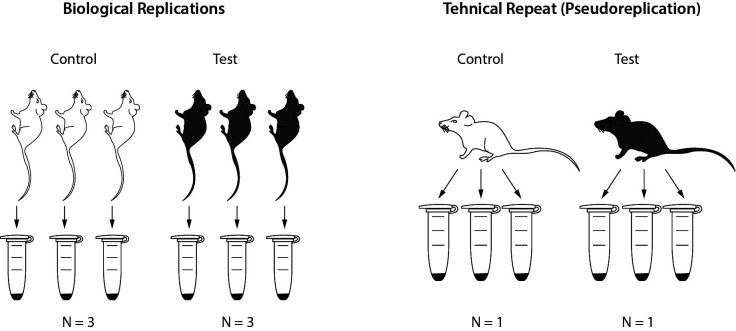
Technical *vs* biological repeat.

Another important point regarding animal studies is the use of technical repetition (pseudo replication) instead of biological repetition. Technical repetition is a specific type of repetition where the same sample is measured multiple times, aiming to probe the noise associated with the measurement method or the device. Here, no matter how many times the same sample is measured, the actual sample size will remain the same. Let us assume a research group is investigating the effect of a therapeutic drug on blood glucose level. If the researchers measure the blood glucose level of 3 mice receiving the actual treatment and 3 mice receiving placebo, this would be a biological repetition. On the other hand, if the blood glucose level of a single mouse receiving the actual treatment and the blood glucose level of a single mouse receiving placebo are each measured 3 times, this would be technical repetition. Both designs will provide 6 data points to calculate P value, yet the P value obtained from the second design would be meaningless since each treatment group will only have one member (Figure 5). Multiple measurements on single mice are pseudo replication; therefore do not contribute to N. No matter how ingenious, no statistical analysis method can fix incorrectly selected replicates at the post-experimental stage; replicate types should be selected accurately at the design stage. This problem is a critical limitation, especially in pre-clinical studies that conduct cell culture experiments. It is very important for critical assessment and evaluation of the published research results (*51*). This issue is mostly underestimated, concealed or ignored. It is striking that in some publications, the actual sample size is found to be as low as one. Experiments comparing drug treatments in a patient-derived stem cell line are specific examples for this situation. Although there may be many technical replications for such experiments and the experiment can be repeated several times, the original patient is a single biological entity. Similarly, when six metatarsals are harvested from the front paws of a single mouse and cultured as six individual cultures, another pseudo replication is practiced where the sample size is actually 1, instead of 6 (*52*). Lazic *et al*. suggested that almost half of the studies (46%) had mistaken pseudo replication (technical repeat) for genuine replication, while 32% did not provide sufficient information to enable evaluation of appropriateness of the sample size (*53*, *54*).

In studies providing qualitative data (such as electrophoresis, histology, chromatography, electron microscopy), the number of replications (“number of repeats” or “sample size”) should explicitly be stated.

Especially in pre-clinical studies, standard error of the mean (SEM) is frequently used instead of SD in some situations and by certain journals. The SEM is calculated by dividing the SD by the square root of the sample size (N). The SEM will indicate how variable the mean will be if the whole study is repeated many times. Whereas the SD is a measure of how scattered the scores within a set of data are. Since SD is usually higher than SEM, researchers tend to use SEM. While SEM is not a distribution criterion; there is a relation between SEM and 95% confidence interval (CI). For example, when N = 3, 95% CI is almost equal to mean ± 4 SEM, but when N ≥ 10; 95% CI equals to mean ± 2 SEM. Standard deviation and 95% CI can be used to report the statistical analysis results such as variation and precision on the same plot to demonstrate the differences between test groups (*52*, *55*).

Given the attrition and unexpected death risk of the laboratory animals during the study, the researchers are generally recommended to increase the sample size by 10% (*56*).

## Sample size calculation for some genetic studies

Sample size is important for genetic studies as well. In genetic studies, calculation of allele frequencies, calculation of homozygous and heterozygous frequencies based on Hardy-Weinberg principle, natural selection, mutation, genetic drift, association, linkage, segregation, haplotype analysis are carried out by means of probability and statistical models (*57*-*62*). While G-Power is useful for basic statistics, substantial amount of analyses can be conducted using genetic power calculator (http://zzz.bwh.harvard.edu/gpc/) (*61*, *62*). This calculator, which provides automated power analysis for variance components (VC) quantitative trait locus (QTL) linkage and association tests in sibships, and other common tests, is significantly effective especially for genetics studies analysing complex diseases.

**Figure 6:**
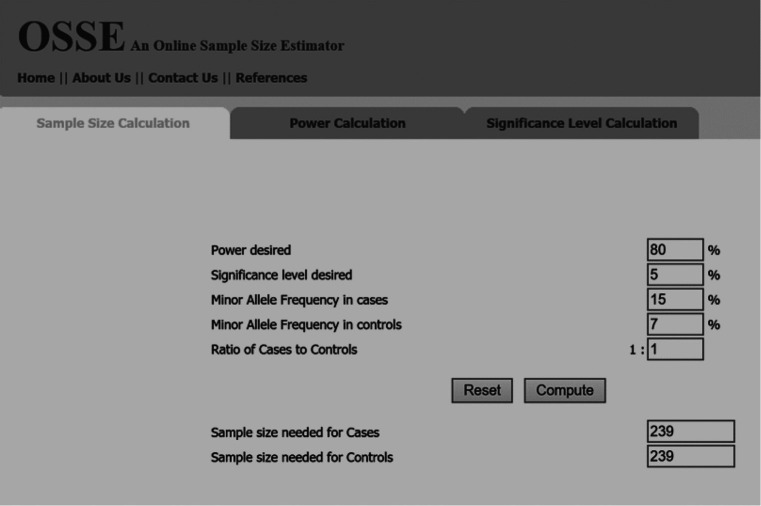
Interface of Online Sample Size Estimator (OSSE) Tool. (Available at: http://osse.bii.a-star.edu.sg/).

Case-control association studies for single nucleotide polymorphisms (SNPs) may be facilitated using OSSE web site (http://osse.bii.a-star.edu.sg/). As an example, let us assume the minor allele frequencies of an SNP in cases and controls are approximately 15% and 7% respectively. To have a power of 0.8 with 0.05 significance, the study is required to include 239 samples both for cases and controls, adding up to 578 samples in total (Figure 6).

Hong and Park have proposed tables and graphics in their article for facilitating sample size estimation (*57*). With the assumption of 5% disease prevalence, 5% minor allele frequency and complete linkage disequilibrium (D’ = 1), the sample size in a case-control study with a single SNP marker, 1:1 case-to-control ratio, 0.8 statistical power, and 5% type I error rate can be calculated according to the genetic models of inheritance (allelic, additive, dominant, recessive, and co-dominant models) and the odd ratios of heterozygotes/rare homozygotes (Table 6). As demonstrated by Hong and Park among all other types of inheritance, dominant inheritance requires the lowest sample size to achieve 0.8 statistical power. Whereas, testing a single SNP in a recessive inheritance model requires a very large sample size even with a high homozygote ratio, that is practically challenging with a limited budget (*57*). The Table 6 illustrates the difficulty in detecting a disease allele following a recessive mode of inheritance with moderate sample size.

**Table 6:** Number of cases required to achieve 0.8 power according to the different genetic models and various odd ratios of heterozygotes/rare homozygotes (OR_het_/OR_homo_) in case-control studies

**Genetic Model**	**OR_het_/OR_homo_ ratio**			
	**1.3/1**	**1.5/1**	**2/3**	**2.5/4**
	**Sample size**			
Allelic	1974	789	248	134
Dominant	606	258	90	53
Co-Dominant	2418	964	301	161
Recessive	20,294	8390	2776	1536
Effective sample sizes are calculated according to the following assumptions: minor allele frequency is 5%, disease prevalence is 5%, there is complete linkage disequilibrium (D’ = 1), case-to-control ratio is 1:1, and the type I error rate is 5% for single marker analysis (57).				

## Sample size and power analyses in clinical studies

In clinical research, sample size is calculated in line with the hypothesis and study design. The cross-over study design and parallel study design apply different approaches for sample size estimation. Unlike pre-clinical studies, a significant number of clinical journals necessitate sample size estimation for clinical studies.

**Figure 7:**
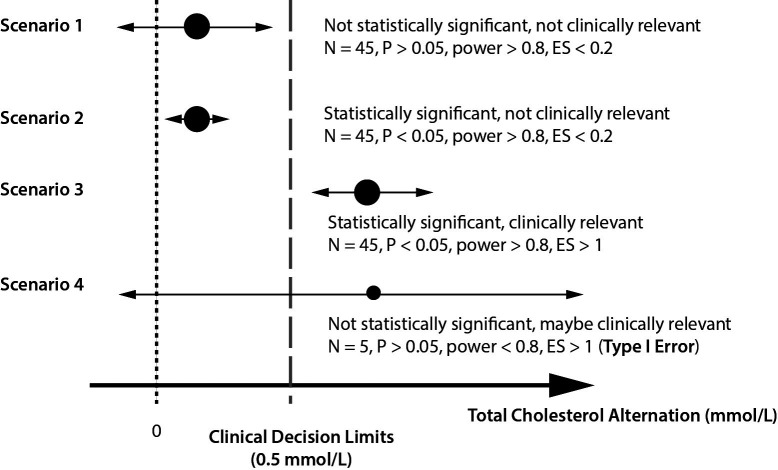
The relationship among clinical significance, statistical significance, power and effect size. In the example above, in order to provide a clinically significant effect, a treatment is required to trigger at least 0.5 mmol/L decreases in cholesterol levels. Four different scenarios are given for a candidate treatment, each having different mean total cholesterol change and 95% confidence interval. ES - effect size. N – number of participant. Adapted from reference 65.
**Similarity and equivalence:** The sample size required demonstrating similarity and equivalence is very low.

The basic rules for sample size estimation in clinical trials are as follows (*63*, *64*):

**Error level (alpha):** It is generally set as < 0.05. The sample size should be increased to compensate for the decrease in the effect size.
**Power** must be **> 0.8:** The sample size should be increased to increase the power of the study. The higher the power, the lower the risk of missing an actual effect.
**The clinical significance**: There is an inverse correlation between the difference in the effect size and the required sample size. To detect smaller differences in the clinical effect, larger sample size is needed and *vice versa*. The clinical significance should be evaluated with effect size, confidence interval, and P value (Figure 7) (*65*).

Sample size estimation can be performed manually using the formulas in Table 1 as well as software and websites in Table 2 (especially by G-Power). However, all of these calculations require preliminary results or previous study outputs regarding the hypothesis of interest. Sample size estimations are difficult in complex or mixed study designs. In addition: a) unplanned interim analysis, b) planned interim analysis and

- adjustments for common variables may be required for sample size estimation.

In addition, *post-hoc* power analysis (possible with G-Power, PASS) following the study significantly facilitates the evaluation of the results in clinical studies.

A number of high-quality journals emphasize that the statistical significance is not sufficient on its own. In fact, they would require evaluation of the results in terms of effect size and clinical effect as well as statistical significance.

In order to fully comprehend the effect size, it would be useful to know the study design in detail and evaluate the effect size with respect to the type of the statistical tests conducted as provided in Table 3.

Hence, the sample size is one of the critical steps in planning clinical trials, and any negligence or shortcomings in its estimate may lead to rejection of an effective drug, process, or marker. Since statistical concepts have crucial roles in calculating the sample size, sufficient statistical expertise is of paramount importance for these vital studies.

## Sample size, effect size and power calculation in laboratory studies

In clinical laboratories, software such as G-Power, Medcalc, Minitab, and Stata can be used for group comparisons (such as t-tests, Mann Whitney U, Wilcoxon, ANOVA, Friedman, Chi-square, *etc.*), correlation analyses (Pearson, Spearman, *etc*.) and regression analyses.

Effect size that can be calculated according to the methods mentioned in Table 3 is important in clinical laboratories as well. However, there are additional important criteria that must be considered while investigating differences or relationships. Especially the guidelines (such as CLSI, RiliBÄK, CLIA, ISO documents) that were established according to many years of experience, and results obtained from biological variation studies provide us with essential information and critical values primarily on effect size and sometimes on sample size.

Furthermore, in addition to the statistical significance (P value interpretation), different evaluation criteria are also important for the assessment of the effect size. These include precision, accuracy, coefficient of variation (CV), standard deviation, total allowable error, bias, biological variation, and standard deviation index, *etc*. as recommended and elaborated by various guidelines and reference literature (*66*-*70*).

In this section, we will assess sample size, effect size, and power for some analysis types used in clinical laboratories.

## Sample size in method and device comparisons

Sample size is a critical determinant for Linear, Passing Bablok, and Deming regression studies that are predominantly being used in method comparison studies. Sample size estimations for the Passing-Bablok and Deming method comparison studies are exemplified in Table 7 and Table 8 respectively. As seen in these tables, sample size estimations are based on slope, analytical precision (% CV), and range ratio (c) value (*66*, *67*). These tables might seem quite complicated for some researchers that are not familiar with statistics. Therefore, in order to further simplify sample size estimation; reference documents and guidelines have been prepared and published. As stated in CLSI EP09-A3 guideline, the general recommendation for the minimum sample size for validation studies to be conducted by the manufacturer is 100; while the minimum sample size for user-conducted verification is 40 (*68*). In addition, these documents clearly explain the requirements that should be considered while collecting the samples for method/device comparison studies. For instance, samples should be homogeneously dispersed covering the whole detection range. Hence, it should be kept in mind that randomly selected 40-100 sample will not be sufficient for impeccable method comparison (*68*).

**Table 7:** Proposed sample size sizes for Passing Bablok regression, (power at least 0.8, alpha = 0.05) (Simplified from reference 66)

		Slope*	**1.00-1.02**		**1.02-1.04**		**1.04-1.06**	**1.06-1.08**		**1.08-1.10**	**1.10-1.12**		**1.12-1.15**		**1.15-1.2**
			**1.00-0.98**		**0.98-0.96**		**0.96-0.94**	**0.94-0.93**		**0.93-0.91**	**0.91-0.89**		**0.89-0.85**		**0.85-0.83**
**Range ratio**	**%CV**	**Proposed Sample Sizes**													
**∞**	2			> 90		30	< 30		< 30	< 30		< 30		< 30	< 30
** **	5			> 90		> 90	80		45	35		< 30		< 30	< 30
	7			> 90		> 90	> 90		90	60		45		30	< 30
** **	10			> 90		> 90	> 90		> 90	> 90		80		55	35
** **	13			> 90		> 90	> 90		> 90	> 90		> 90		80	50
** 4**	2			> 90		90	40		< 30	< 30		< 30		< 30	< 30
** **	5			> 90		> 90	> 90		> 90	85		65		40	< 30
	7			> 90		> 90	> 90		> 90	> 90		> 90		80	45
** **	10			> 90		> 90	> 90		> 90	> 90		> 90		> 90	80
**2**	2			> 90		> 90	> 90		75	50		35		< 30	< 30
** **	5			> 90		> 90	> 90		> 90	> 90		> 90		> 90	80
Slope - the steepness of a line and the intercept indicates the location where it intersects an axis. The greater the magnitude of the slope, the steeper the line and the greater the rate of change. The formula for the regression line in method comparison study is y = ax + b, where a is the slope of the line and b is the y-intercept. The range ratio (concentration of the upper limit / concentration of the lower limit). % CV - coefficient of variation (analytical precision). *Sample size values are proposed for respective slope ranges. i.e. for range ratio: 4, CV: 2%, slope range: 1.00–1.02 or 1.00–0.98 requires > 90 samples; whereas slope range: 1.04-1.06 or 0.96-0.94 requires 40 samples. Note: In this example, similar % CV values are assumed for the two methods compared. For methods having dissimilar % CV values, the researcher should refer to the reference 66.															

**Table 8:** Necessary sample sizes for test of slope deviation from 1 or intercept deviation from zero by Deming and Weighted regression analysis

**Standardized Δ value for slope**											
**In Deming regression**	**Range ratio**	**1.25**	**1.5**	**2**	**2.5**	**3**	**4**		**5**	**8**	**10**
		**Proposed Sample Size**									
**1**		5104	1575	567	343	256	182		150	116	108
**2**		1276	410	152	90	69	48		39	32	27
**3**		585	185	70	42	32	25		20	16	15
**4**		325	104	41	27	20	15		13	11	≤ 10
**In weighted Deming Regression**	**Range ratio**	**2**	**2.5**	**3**	**4**	**5**	**8**		**10**	**25**	**50**
		**Proposed Sample Size**									
**1**		544	320	226	150	114	75	64		45	37
**2**		144	82	61	40	33	23	20		18	15
**3**		66	42	29	22	17	≤ 10	≤ 10		≤ 10	≤ 10
**4**		39	26	19	15	12	≤ 10	≤ 10		≤ 10	≤ 10
Type I error = 0.05. Power = 0.9. Standardized Δ value for slope *= (*Slope *- 1) / CV*. CV – coefficient of variation. The range ratio - concentration of the upper limit / concentration of the lower limit. CV refers to the CV at the middle of the given interval (SD / mean of the interval for the analytes), *i.e.* while the required sample size is 343 for a “standardized Δ value for slope” of 1 for a range ratio of 2.5 in Deming regression, it is 320 in weighted Deming regression (Simplified from reference 66).											

Additionally, comparison studies might be carried out in clinical laboratories for other purposes; such as inter-device, where usage of relatively few samples is suggested to be sufficient. For method comparison studies to be conducted using patient samples; sample size estimation, and power analysis methodologies, in addition to the required number of replicates are defined in CLSI document EP31-A-IR. The critical point here is to know the values of constant difference, within-run standard deviation, and total sample standard deviation (*69*). While studies that compare devices having high analytical performance would suffice lower sample size; studies comparing devices with lower analytical performance would require higher sample size.

Lu *et al.* used maximum allowed differences for calculating sample sizes that would be required in Bland Altman comparison studies. This type of sample size estimation, which is critically important in laboratory medicine, can easily be performed using Medcalc software (*70*).

## Sample size in lot to lot variation studies

It is acknowledged that lot-to-lot variation may influence the test results. In line with this, method comparison is also recommended to monitor the performance of the kit in use, between lot changes. To aid in the sample size estimation of these studies; CLSI has prepared the EP26-A guideline “User evaluation of between-reagent lot variation; approved guideline”, which provides a methodology like EP31-A-IR (*71*, *72*).

The Table 9 presents sample size and power values of a lot-to-lot variation study comparing glucose measurements at 3 different concentrations. In this example, if the difference in the glucose values measured by different lots is > 0.2 mmol/L, > 0.58 mmol/L and > 1.16 mmol/L at analyte concentrations of 2.77 mmol/L, 8.32 mmol/L and 16.65 mmol/L respectively, lots would be confirmed to be different. In a scenario where one sample is used for each concentration; if the lot-to-lot variation results obtained from each of the three different concentrations are lower than the rejection limits (meaning that the precision values for the tested lots are within the acceptance limits), then the lot variation is accepted to lie within the acceptance range. While the example for glucose measurements presented in the guideline suggests that “1 sample” would be sufficient at each analyte concentration, it should be noted that sample size might vary according to the number to devices to be tested, analytical performance results of the devices (*i.e.* precision), total allowable error, *etc.* For different analytes and scenarios (*i.e.* for occasions where one sample/concentration is not sufficient), researchers need to refer CLSI EP26-A (*71*).

**Table 9:** Sample size and power values of a lot-to-lot variation studies

**Analyte**	**Target concentration** **(mmol/L)**	**Cd**	**S_wrl_**	**S_r_**	**Cd/S_wrl_**	**S_r_/S_wrl_**	**Rejection limit** **(mmol/L)**	**Sample Size (N)**	**Power**
Glucose	2.77	0.33	0.055	0.033	6.0	0.6	0.6 x Cd (0.2)	1	0.955
	8.32	0.83	0.11	0.08	7.5	0.75	0.7 x Cd (0.58)	1	> 0.916
	16.65	1.66	0.25	0.19	6.7	0.78	0.7 x Cd (1.16)	1	> 0.916
Cd - critical difference is the total allowable error (TAE) according to the CLIA criteria. S_r_ - repeatability (within-run imprecision). S_wrl_ - within-reagent lot imprecision. Note: S_r_ and S_wrl_ values should be obtained from the manufacturer. Power is calculated according to critical difference, imprecision values and sample size as explained in detail in CLSI EP 26-A. If the lot-to-lot variation results obtained from three different concentrations are lower than the rejection limits when one sample is used for each concentration (meaning method precision of the tested lots are within the acceptance limits), then the lot variation is said to remain within the acceptance range. (The actual table provided in the guideline (CSLI EP26A) is of 3 pages. Since the primary aim of this paper is to familiarize the reader with sample size estimation methodologies in different study types; for simplification, only a glucose example is included in this table. For different analytes and scenarios (*i.e.* for occasions where one sample/concentration is not sufficient), researchers need to refer CLSI EP26-A.) (71).									

Some researchers find CLSI EP26-A and CLSI EP31 rather complicated for estimating the sample size in lot-to-lot variation and method comparison studies (which are similar to a certain extent). They instead prefer to use the sample size (number of replicates) suggested by Mayo Laboratories. Mayo Laboratories decided that lot-to-lot variation studies may be conducted using 20 human samples where the data are analysed by Passing-Bablok regression and accepted according to the following criteria: a) slope of the regression line will lie between 0.9 and 1.1; b) R2 coefficient of determination will be > 0.95; c) the Y-intercept of the regression line will be < 50% of the lowest reportable concentration, d) difference of the means between reagent lots will be < 10% (*73*).

## Sample size in verification studies

Acceptance limits should be defined before the verification and validation studies. These could be determined according to clinical cut-off values, biological variation, CLIA criteria, RiliBÄK criteria, criteria defined by the manufacturer, or state of the art criteria. In verification studies, the “sample size” and the “minimum proportion of the observed samples required to lie within the CI limits” are proportional. For instance, for a 50-sample study, 90% of the samples are required to lie within the CI limits for approval of the verification; while for a 200-sample study, 93% is required (Table 10). In an example study whose total allowable error (TAE) is specified as 15%; 50 samples were measured. Results of the 46 samples (92% of all samples) lied within the TAE limit of 15%. Since the proportion of the samples having results within the 15% TAE limit (92% of the samples) exceeds the minimum proportion required to lie within the TAE limits (90% of the samples), the method is verified (*74*).

**Table 10:** Sample size estimation in method verification studies

**N**	**Minimum percentage of the observed samples required to lie within the CI limits (%)**
20	85
30	87
40	90
50	90
100	91
200	93
500	93
1000	94
N – sample size. CI – confidence interval. *I.e.* for a verification study of 20 samples, 85% of the samples (17 samples) are required to lie within the CI limits, whereas for a verification study of 100 samples, 91% of the samples (91 samples) are required to lie within the CI limits (74).	

Especially in recent years, researchers tend to use CLSI EP15-A3 or alternative strategies relying on EP15-A3, for verification analyses. While the alternative strategies diverge from each other in many ways, most of them necessitate a sample size of at least 20 (*75*-*78*). Yet, for bias studies, especially for the ones involving External Quality Control materials, even lower sample sizes (*i.e.* 10) may be observed (*79*). Verification still remains to be one of the critical problems for clinical laboratories. It is not possible to find a single criteria and a single verification method that fits all test methods (*i.e.* immunological, chemical, chromatographical, *etc.*).

While sample size for qualitative laboratory tests may vary according to the reference literature and the experimental context, CLSI EP12 recommends at least 50 positive and 50 negative samples, where 20% of the samples from each group are required to fall within cut-off value +/- 20% (*80*, *81*). According to the clinical microbiology validation/verification guideline Cumitech 31A, the minimum number of the samples in positive and negative groups is 100/each group for validation studies, and 10/each group for verification studies (*82*).

## Sample size in diagnostic and prognostic studies

ROC analysis is the most important statistical analysis in diagnostic and prognostic studies. Although sample size estimation for ROC analyses might be slightly complicated; Medcalc, PASS, and Stata may be used to facilitate the estimation process. Before the actual size estimations, it is a prerequisite for the researcher to calculate potential area under the curve (AUC) using data from previous or preliminary studies. In addition, size estimation may also be calculated manually according to Table 1, or using sensitivity (or TPF) and 1-specificity (FPF) values according to Table 11 which is adapted from CLSI EP24-A2 (*83*, *84*).

**Table 11:** Determining sample size in diagnostic studies

**Sensitivity or Specificity (TPF or 1-FPF)**	**L**	**N**
0.80	0.05	246
0.85	0.05	196
0.90	0.05	139
0.95	0.05	73
0.70	0.10	81
0.75	0.10	73
0.80	0.10	62
0.85	0.10	49
L - desired width of one half of the confidence interval (CI), or maximum allowable error of the estimate. (95% CI for 0.05 and 90% CI for 0.10). TPF - true positive fraction. FPF - false positive fraction. Adapted from CLSI EP24-A2, reference 83.		

As is known, X-axis of the ROC curve is FPF, and Y-axis is TPF. While TPF represents sensitivity, FPF represents 1-specificity. Utilizing Table 11, for a 0.85 sensitivity, 0.90 specificity and a maximum allowable error of 5% (L = 0.05), 196 positive and 139 negative samples are required. For the scenarios not included in this table, reader should refer to the formulas given under “diagnostic prognostic studies” subsection of Table 1.

Standards for reporting of diagnostic accuracy studies (STARD) checklist may be followed for diagnostic studies. It is a powerful checklist whose application is explained in detail by Cohen *et al.* and Flaubaut *et al.* (*85*, *86*). This document suggests that, readers demand to understand the anticipated precision and power of the study and whether authors were successful in recruiting the sufficient number of participants; therefore it is critical for the authors to explain the intended sample size of their study and how it was determined. For this reason, in diagnostic and prognostic studies, sample size and power should clearly be stated.

As can be seen here, the critical parameters for sample size estimation are AUC, specificity and sensitivity, and their 95% CI values. The table 12 demonstrates the relationship of sample size with sensitivity, specificity, negative predictive value (NPV) and positive predictive value (PPV); the lower the sample size, the higher is the 95% CI values, leading to increase in type II errors (*87*). As can be seen here, confidence interval is narrowed as the sample size increases, leading to a decrease in type II errors.

**Table 12:** Relationship between sample size and 95% CI of a test characteristic (sensitivity, specificity, positive predictive value (PPV), negative predictive value (NPV), ratio of false-positives (FPR) and ratio of false-negatives (FNR) *etc*; are ratios between 0.00–1.00)

**Sample size**	**95% CI** **for a ratio of 0.05** **(*i.e.* FPR = 0.05, FNR = 0.05, *etc*.)**	**95% CI** **for a ratio of 0.80** **(*i.e.* sensitivity = 0.80, specificity = 0.80, PPV = 0.80, NPV = 0.80, *etc*.)**
20	0.00-0.25	0.56-0.94
60	0.01-0.14	0.68-0.90
100	0.02-0.11	0.71-0.87
500	0.03-0.07	0.76-0.83
1000	0.04-0.07	0.77-0.82
95% CI of the test characteristic ratios of 0.05 and 0.8 are selected for illustration. Test characteristics such as sensitivity, specificity, positive predictive value, negative predictive value, false-positives and false-negatives are denoted either as percentages or ratios. To use a terminology similar to the original table, the term “ratio” is preferred here. The 95% CI is inversely proportional with the sample size; 95% CI is narrower with increased sample size. In the example here, a diagnostic study having a sensitivity of 0.8 is provided. The 95% CI is broader (0.56–0.94) if the study is conducted with 20 samples, and narrower (0.71–0.87) is the study is conducted with 100 samples. Thus, at small sample sizes, only rather uncertain estimates of specificity, sensitivity, FPR, FNR, *etc.* are obtained (87).		

Like all sample size calculations, preliminary information is required for sample size estimations in diagnostic and prognostic studies. Yet, variation occurs among sample size estimates that are calculated according to different reference literature or guidelines. This variation is especially prominent depending on the specific requirements of different countries and local authorities.

While sample size calculations for ROC analyses may easily be performed *via* Medcalc, the method explained by Hanley *et al.* and Delong *et al.* may be utilized to calculate sample size in studies comparing different ROC curves (*88*, *89*).

## Sample size for reference interval determination

Both IFCC working groups and the CLSI guideline C28-A3c offer suggestions regarding sample size estimations in reference interval studies (*90*-*93*). These references mainly suggest at least 120 samples should be included for each study sub-group (*i.e.,* age-group, gender, race, *etc.*). In addition, the guideline also states that, at least 20 samples should be studied for verification of the determined reference intervals.

Since extremes of the observed values may under/over-represent the actual percentile values of a population in nonparametric studies, care should be taken not to rely solely on the extreme values while determining the nonparametric 95% reference interval. Reed *et al.* suggested a minimum sample size of 120 to be used for 90% CI, 146 for 95% CI, and 210 for 99% CI (93). Linnet proposed that up to 700 samples should be obtained for results having highly skewed distributions (*94*). The IFCC Committee on Reference Intervals and Decision Limits working group recommends a minimum of 120 reference subjects for nonparametric methods, to obtain results within 90% CI limits (*90*).

Due to the inconvenience of the direct method, in addition to the challenges encountered using paediatric and geriatric samples as well as the samples obtained from complex biological fluids (*i.e.* cerebrospinal fluid); indirect sample size estimations using patient results has gained significant importance in recent years. Hoffmann method, Bhattacharya method or their modified versions may be used for indirect determination of the reference intervals (*95*-*101*). While a specific sample size is not established, sample size between 1000 and 10.000 is recommended for each sub-group. For samples that cannot be easily acquired (*i.e.* paediatric and geriatric samples, and complex biological fluids), sample sizes as low as 400 may be used for each sub-group (*92*, *100*).

## Sample size in survey studies

The formulations given on Table 1 and the websites mentioned on Table 2 will be particularly useful for sample size estimations in survey studies which are dependent primarily on the population size (*101*).

Three critical aspects should be determined for sample size determination in survey studies:

1. Population size
2. Margin of Error (ME) is predominantly important for survey studies. The ME expresses the amount of random sampling error in survey results. Larger margin of error would suggest that the poll results are less likely to reflect the survey results of an entire population. Table 13 may provide a practical solution for size estimation. A 5% ME means that, the actual population value is expected to lie within survey result ± 5%. 1-10% is selected as margin of error in general. The ME above 10% is not recommended. It is possible to calculate ME% using the following formula, ME% = 100 / √N. For instance, while ME% will be 31.6% for a sample size of 10 (ME% = 100 / √10 = 31.6), it will be 3.16% for a sample size of 1000 (ME% = 100 / √1000 = 3.16). The ME above 10% is not recommended (*102*).

**Table 13:** Sample size estimation according to the population size (merely as rough estimates), margin of error (ME) and confidence interval (CI)

		**Margin of error (ME) ** **(for CI 95%)**				**Confidence Interval (CI) (for ME 5%)**		
**Population Size**	**10%**	**5%**	**1%**	**90%**	**95%**		**99%**	
100	50	80	99	74	80		88	
500	81	218	476	176	218		286	
1000	88	278	906	215	278		400	
10,000	96	370	4900	264	370		623	
100,000	96	383	8763	270	383		660	
1.000,000	97	384	9513	271	384		664	
Sample size estimation may be performed according to the actual population size, margin of error and confidence interval. Here most commonly used ME (5%) and CI (95%) levels are exemplified. A variation in ME causes a more drastic change in sample size than a variation in CI. As an example, for a population of 10,000 people, a survey with a 95% CI and 5% ME would require at least 370 samples. When CI is changed from 95% to 90% or 99%, the sample size which was 370 initially would change into 264 or 623 respectively. Whereas, when ME is changed from 5% to 10% or 1%; the sample size which was initially 370 would change into 96 or 4900 respectively. For other ME and CI levels, the researcher should refer to the equations and software provided on Table 1 and Table 2 (102).
3. Confidence Interval (CI) of 95% means that, when the study is repeated, with 95% probability, the same results will be obtained. Depending on the hypothesis and the study aim, confidence interval may lie between 90% and 99%. Confidence interval below 90% is not recommended.

For a given CI, sample size and ME is inversely proportional; sample size should be increased in order to obtain a narrower ME. On the contrary, for a fixed ME, CI and sample size is directly proportional; in order to obtain a higher CI, the sample size should be increased. In addition, sample size is directly proportional to the population size; higher sample size should be used for a larger population. A variation in ME causes a more drastic change in sample size than a variation in CI. As exemplified in Table 13, for a population of 10,000 people, a survey with a 95% CI and 5% ME would require at least 370 samples. When CI is changed from 95% to 90% or 99%, the sample size which was 370 initially would change into 264 or 623 respectively. Whereas, when ME is changed from 5% to 10% or 1%; the sample size which was initially 370 would change into 96 or 4900 respectively. For other ME and CI levels, the researcher should refer to the equations and software provided on Table 1 and Table 2.

The situation is slightly different for the survey studies to be conducted for problem detection. It would be most appropriate to perform a preliminary survey with a small sample size, followed by a power analysis, and completion of the study using the appropriate number of samples estimated based on the power analysis. While 30 is suggested as a minimum sample size for the preliminary studies, the optimal sample size can be determined using the formula suggested in Table 14 which is based on the prevalence value (*103*). It is unlikely to reach a sufficient power for revealing of uncommon problems (prevalence 0.02) at small sample sizes. As can be seen on the table, in the case of 0.02 prevalence, a sample size of 30 would yield a power of 0.45. In contrast, frequent problems (*i.e.* prevalence 0.30) were discovered with higher power (0.83) even when the sample size was as low as 5. For situations where power and prevalence are known, effective sample size can easily be estimated using the formula in Table 1.

**Table 14:** The relation among prevalence, sample size and power of a study that will detect a problem after "N" number of interviews

	**Power values, for a given sample size (number of interview) (N)**						
**Prevalence**	**N = 5**	**N = 7**	**N = 10**	**N = 15**	**N = 20**	**N = 30**	**N = 50**
0.01	0.05	0.07	0.1	0.14	0.18	0.26	0.39
0.02	0.1	0.13	0.18	0.26	0.33	0.45	0.64
0.03	0.14	0.19	0.26	0.37	0.46	0.6	0.78
0.04	0.18	0.25	0.34	0.46	0.56	0.71	0.87
0.05	0.23	0.3	0.4	0.54	0.64	0.79	0.92
0.10	0.41	0.52	0.65	0.79	0.88	0.96	> 0.99
0.15	0.56	0.68	0.8	0.91	0.96	> 0.99	> 0.99
0.20	0.67	0.79	0.89	0.96	0.99	> 0.99	> 0.99
0.25	0.76	0.87	0.94	0.99	> 0.99	> 0.99	> 0.99
0.30	0.83	0.92	0.97	> 0.99	> 0.99	> 0.99	> 0.99
When prevalence is low, higher sample size is required to reach sufficient power. I.e. for a prevalence of 0.2, even 10 interviews (N = 10) is sufficient to reach a power value of 0.89. However, for a prevalence of 0.05, with 10 interviews (N = 10) the power will remain at 0.4, leading to a type II error. According to reference 103.							

## Does big sample size always increase the impact of a study?

While larger sample size may provide researchers with great opportunities, it may create problems in interpretation of statistical significance and clinical impact. Especially in studies with big sample sizes, it is critically important for the researchers not to rely only on the magnitude of the regression (or correlation) coefficient, and the P value. The study results should be evaluated together with the effect size, study efficiencies (*i.e.* basic research, clinical laboratory, and clinical studies) and confidence interval levels. Monte Carlo simulations could be utilized for statistical evaluations of the big data results (*18*, *104*).

As a result, sample size estimation is a critical step for scientific studies and may show significant differences according to research types. It is important that sample size estimation is planned ahead of the study, and may be performed through various routes:

If a similar previous study is available, or preliminary results of the current study are present, their results may be used for sample size estimations *via* the websites and software mentioned in Table 1 and Table 2. Some of these software may also be used to calculate effect size and power.
If the magnitude of the measurand variation that is required for a substantial clinical effect is available (*i.e.* significant change is 0.51 mmol/L for cholesterol, 26.5 mmol/L for creatinine, *etc.*), it may be used for sample size estimation (Figure 7). Presence of Total Allowable Error, constant and critical differences, biological variations, reference change value (RCV), *etc.* will further aid in sample size estimation process. Free software (especially G-Power) and web sites presented on Table 2 will facilitate calculations.
If effect size can be calculated by a preliminary study, sample size estimations may be performed using the effect size (*via* G-Power, Table 4, *etc.*)
In the absence of a previous study, if a preliminary study cannot be performed, an effect size may be initially estimated and be used for sample size estimations
If none of the above is available or possible, relevant literature may be used for sample size estimation.
For clinical laboratories, especially CLSI documents and guidelines may prove useful for sample size estimation (Table 9,11).

Sample size estimations may be rather complex, requiring advanced knowledge and experience. In order to properly appreciate the concept and perform precise size estimation, one should comprehend properties of different study techniques and relevant statistics to certain extend. To assist researchers in different fields, we aimed to compile useful guidelines, references and practical software for calculating sample size and effect size in various study types. Sample size estimation and the relationship between P value and effect size are key points for comprehension and evaluation of biological studies. Evaluation of statistical significance together with the effect size is critical for both basic science, and clinical and laboratory studies. Therefore, effect size and confidence intervals should definitely be provided and its impact on the laboratory/clinical results should be discussed thoroughly.
